# HIV-1 Gp120 Protein Downregulates Nef Induced IL-6 Release in Immature Dentritic Cells through Interplay of DC-SIGN

**DOI:** 10.1371/journal.pone.0059073

**Published:** 2013-03-15

**Authors:** Roni Sarkar, Debashis Mitra, Sekhar Chakrabarti

**Affiliations:** 1 Division of Virology, National Institute of Cholera and Enteric Diseases, Kolkata, India; 2 National Centre for Cell Science, Pune, India; Salute San Raffaele University School of Medicine, Italy

## Abstract

HIV-1 replication is a tightly controlled mechanism which demands the interplay of host as well as viral factors. Both gp120 (envelope glycoprotein) and Nef (regulatory protein) have been correlated with the development of AIDS disease in independent studies. In this context, the ability of HIV-1 to utilize immature dentritic cells for transfer of virus is pivotal for early pathogenesis. The presence of C-type lectins on dendritic cells (DCs) like DC-SIGN, are crucial in inducing antiviral immunity to HIV-1. Both gp120 and Nef induce the release of cytokines leading to multiple effects of viral pathogenesis. Our study elucidated for the first time the cross-talk of the signaling mechanism of these two viral proteins in immature monocyte derived dentritic cells (immDCs). Further, gp120 was found to downregulate the IL-6 release by Nef, depending on the interaction with DC-SIGN. A cascade of signaling followed thereafter, including the activation of SOCS-3, to mediate the diminishing effect of gp120. Our results also revealed that the anti-apoptotic signals emanated from Nef was put to halt by gp120 through inhibition of Nef induced STAT3. Thus our results implicate that the signaling generated by gp120 and Nef, undergoes a switch-over mechanism that significantly contributes to the pathogenesis of HIV-1 and widens our view towards the approach on battling the viral infection.

## Introduction

HIV-1 infection is characterized by sustained activation of the immune system. Dendritic cells are the first to encounter HIV-1 in the mucosal genital tissues [1] and are positioned throughout the peripheral immune system [2]–[4]. DCs are crucial for initiating immune response due to their ability to present processed antigens to recirculating naïve T cells [5] after migrating to secondary lymphoid organs. The initial step in HIV-1 infection occurs by binding of the viral envelope glycoprotein gp120 to surface CD4 followed by interactions with CCR5 or both in T lymphocytes and macrophages [6]. However, it has been seen that HIV-1 can efficiently bind to receptors apart from CD4 in dendritic cells [7]–[11]. A major C type lectin, DC-SIGN, found on DCs, has been characterized as a gp120 binding protein of affinity higher than that of CD4 [9]. HIV-1 internalization occurs through DC-SIGN in both immature and mature DCs, thus leading to enhanced infection via DC-SIGN within CD4 lymphocytes and through trans-infection [12], [13]. Hence DCs have significant roles in aspects pertaining to innate and adaptive immunological responses.

Based on molecular epidemiological data in humans the HIV-1 regulatory protein, Nef, has a key role to play in viral pathogenesis [14]–[16]. Since animal models stand as evidence for the complete development of AIDS by the presence of Nef [17], [18], hence the study of Nef on DCs appears to be of profound interest. Nef is a 27-kDa protein expressed early in the virus life cycle and modulates several signaling pathways [19]. Exogenous Nef has been detected in the sera of AIDS patients and in cultures of HIV-1 infected cells [20]. Another crucial HIV-1 protein, gp120, which is an envelope glycoprotein, is implicated in signaling mechanism, extensively investigated in dendritic cells. The activation of cellular signaling pathways leading to the production of cytokine and chemokine genes by HIV-1 gp120 could facilitate viral replication in the early phases of the viral life cycle [21]. Hence the impact of the combined study of Nef mediated signaling in presence of gp120 on DCs, is vital to understand the basis of the foundation of HIV-1 pathogenesis.

During the initial phase of HIV-1 infection, primary cytokines such as IL-6, IL-10, TNF-alpha, MIP-1alpha and MIP-1beta enter the scenario [22]–[24]. These cytokines have pivotal roles in the regulation of immune response despite their pleiotropy. HIV-1 hijacks the release mechanism of these cytokines since they serve as the pillars of host immune system during viral entry. Our present study, while estimating the level of these cytokines *in vitro* through stimulation of immature DCs with gp120, Nef and a combination of both the proteins, exhibited a drop in IL-6 titer when brought in comparison to other cytokines. We therefore performed an in-depth study to explore the pathway responsible for this observation.

Our experiments yielded the result where gp120 was found to downregulate Nef dependent IL-6 release by the involvement of SOCS-3, an important intracellular molecule responsible for terminating the damaging effects of cytokines. Several data have showed the roles of individual HIV-1 proteins, gp120 and Nef in the past [19]–[21], [23]–[26]. However, what is not known is the interaction of different signaling pathways initiated by these two proteins. Since immature DCs serve as the target for HIV-1 attack, hence it would be quite interesting to find out how these two proteins act together during HIV-1 infection. Definitely there might to be a switching mechanism between the two during the viral life cycle. Here we have elucidated how immature DCs react in the presence of both gp120 and Nef through a redundant mechanism of cell activation. The consequent release of different activating and inhibiting factors in the process furthermore extends the impact of this crosstalk. Since immature DCs have the unique capacity to act as vehicles for HIV-1 transmission [9], hence we performed our experiments with immDCs to find out the signaling mechanism arising due to the crosstalk of gp120 and Nef. Our study will provide new leads to HIV-1 trafficking through DCs for better understanding of AIDS pathogenesis. Thus we will be able to know how gp120 and Nef enhance viral infection without neutralizing the action of each other.

## Results

### Gp120 downregulates Nef dependent IL-6 release

**Figure 1:**
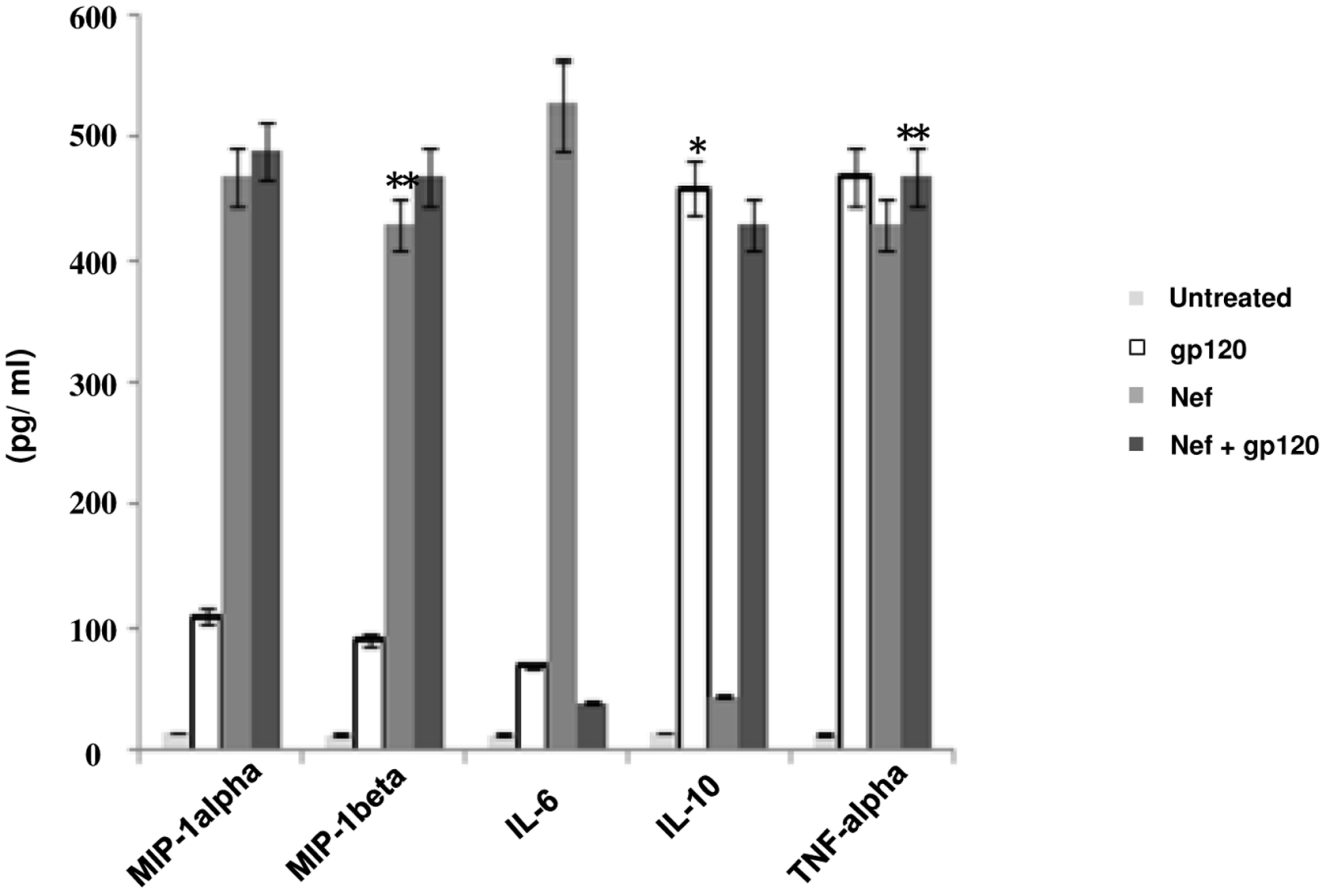
Measurement of cytokines in immDCs. The concentration of various cytokines (MIP- 1alpha, MIP- 1beta, IL-6, IL-10 and TNF- alpha) in the supernatant of immDCs after incubating with either Nef (100 ng/ml), gp120 (4 µg/ml) or a combination of both Nef (100 ng/ml) and gp120 (4 µg/ml) in separate sets of experiments were measured. Data represent the mean±SEM (n = 3). Statistical analysis was performed using Student' t-test, with the levels of significance defined as p*<0.05 and p**<0.01.

**Figure 2:**
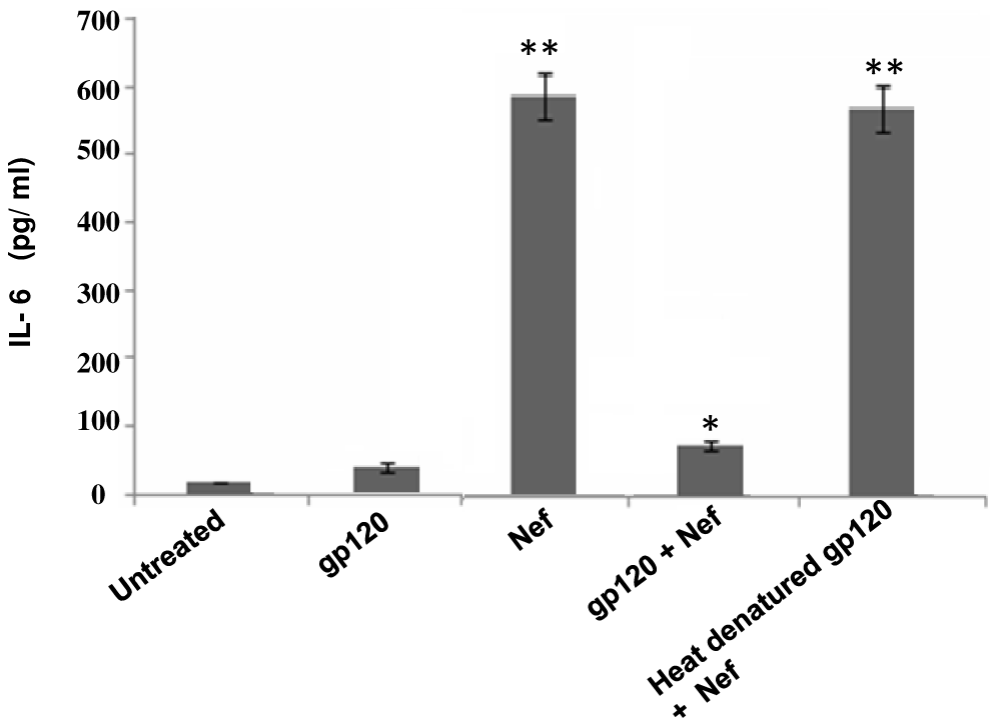
gp120 downregulates Nef dependent IL-6 release. ELISA of IL-6 concentration was measured in pg/ml in immDCs. The first column denotes untreated cells; the second column denotes cells treated with only gp120; the third column denotes cells treated with only Nef; the fourth column denotes cells treated with both gp120 and Nef; the fifth column denotes cells treated with heat denatured gp120 along with Nef. Data represent the mean±SEM (n = 3). Statistical analysis was performed using Student's t-test, with the levels of significance defined as p*<0.05 and p**<0.01.

**Figure 3:**
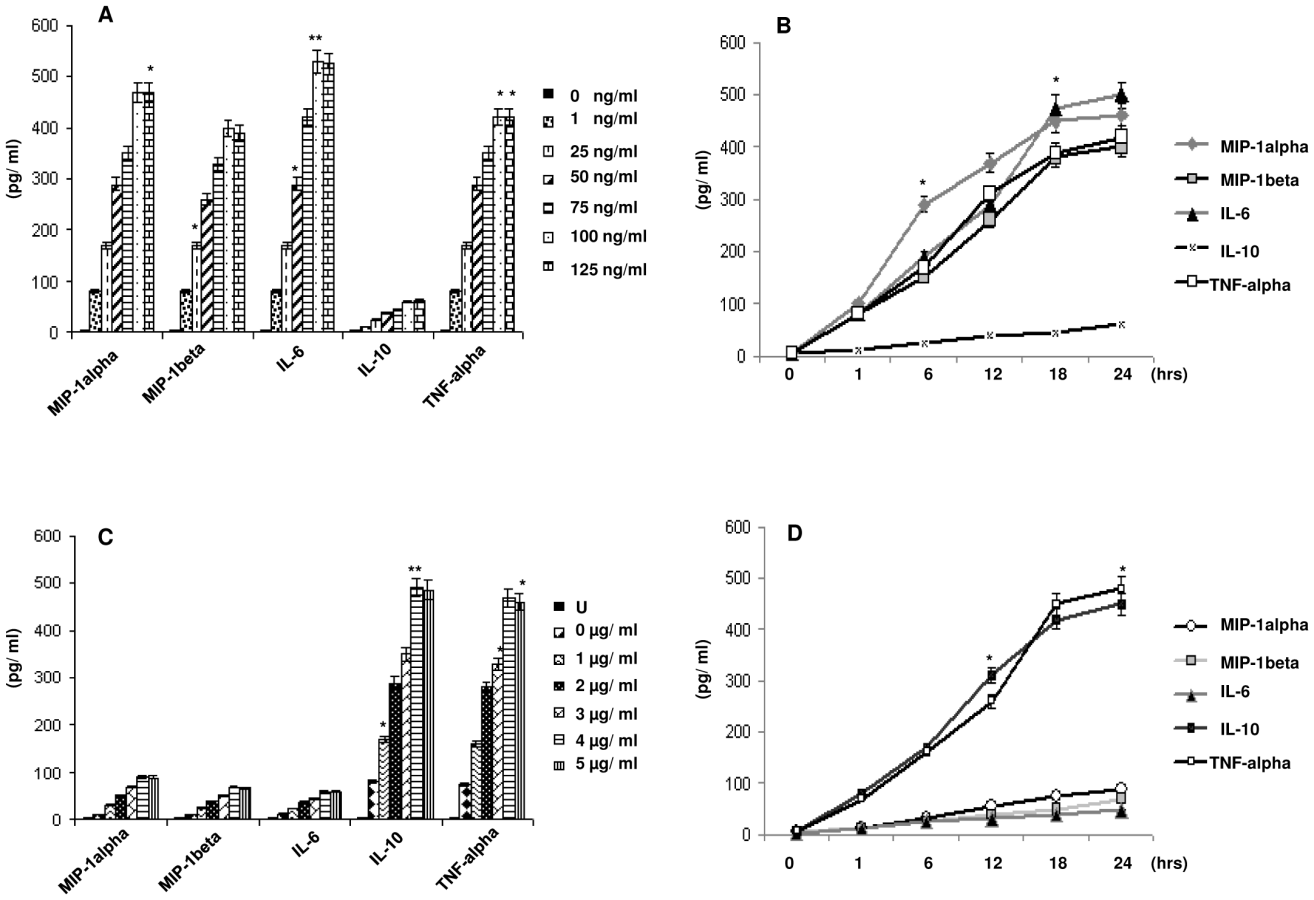
The effect of gp120 and Nef on various cytokines (MIP- 1alpha, MIP- 1beta, IL-6, IL-10 and TNF- alpha) in a dose and time dependent manner. (**A**) The immDCs were incubated in presence of different concentrations of Nef with 0 ng/ml, 1 ng/ml, 25 ng/ml, 50 ng/ml, 75 ng/ml, 100 ng/ml and 125 ng/ml. The supernatant was collected after 24 hrs followed by measurement of cytokine concentrations through ELISA. Data represent the mean±SEM (n = 3). Statistical analysis was performed using Student's t-test, with the levels of significance defined as p*<0.05 and p**<0.01. (**B**) The immDCs were incubated in presence of Nef (100 ng/ml) for upto 24 hrs time. The supernatant was collected and the cytokine concentrations were detected through ELISA. Data represent the mean±SEM (n = 3). Statistical analysis was performed using Student's t-test, with the levels of significance defined as p*<0.05. (**C**) The immDCs were incubated in presence of different concentrations of gp120 (0 µg/ml, 1 µg/ml, 2 µg/ml, 3 µg/ml, 4 µg/ml and 5 µg/ml). The supernatant was collected after 24 hrs followed by measurement of cytokine concentrations through ELISA. Data represent the mean±SEM (n = 3). Statistical analysis was performed using Student's t-test, with the levels of significance defined as p*<0.05 and p**<0.01. (**D**) The immDCs were incubated in presence of gp120 (4 µg/ml) for upto 24 hrs time. The supernatant was collected and the cytokine concentrations were detected through ELISA. Data represent the mean±SEM (n = 3). Statistical analysis was performed using Student's t-test, with the levels of significance defined as p*<0.05.

**Figure 4:**
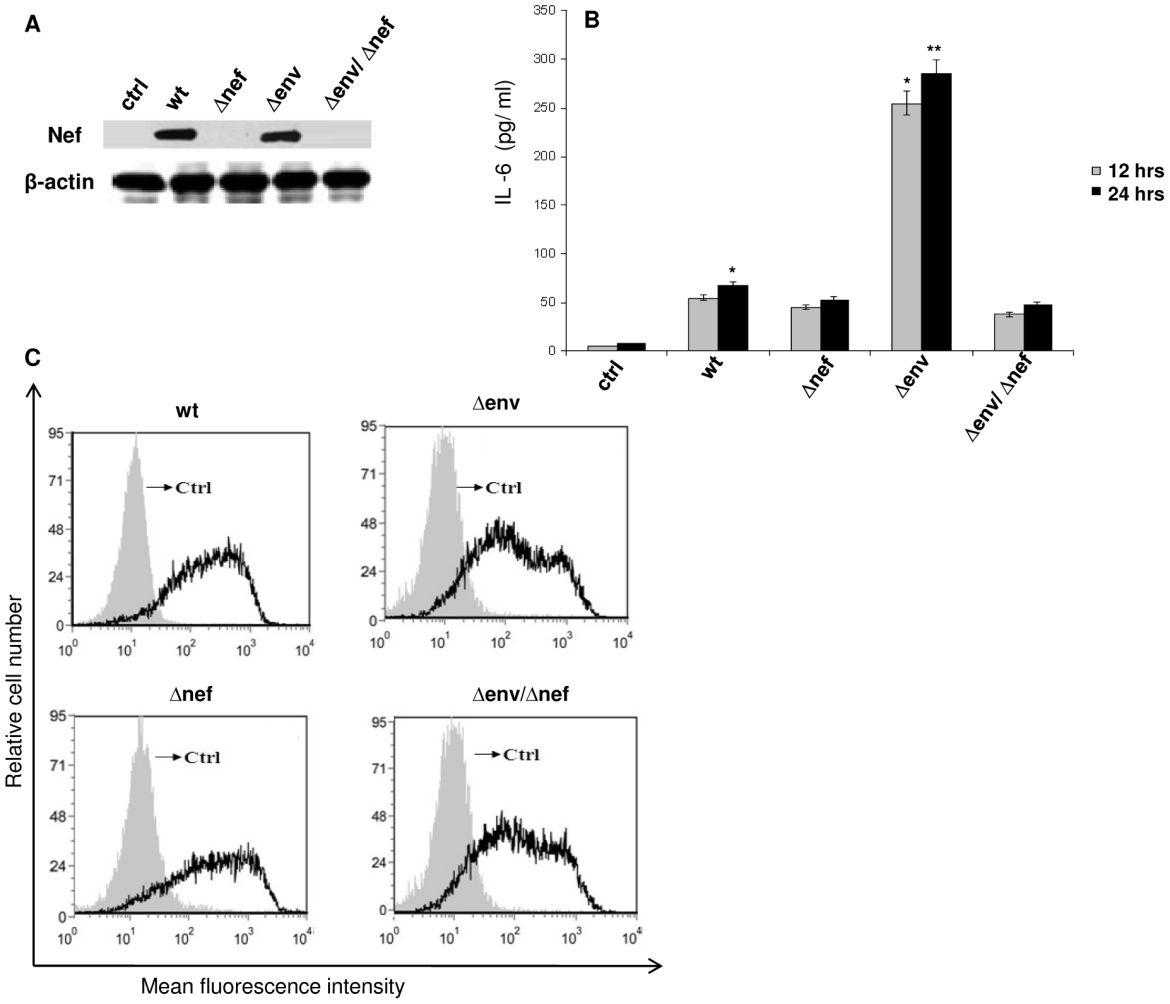
(A) Expression of Nef in the presence of wt, *Änef*, *Äenv*, *Änef*/*Äenv* (VSV-G) HIV-1 or mock infected immDCs. The immDCs were infected with the wild type and mutant viruses. After 24 hrs, the cells were harvested and assayed for Nef expression by immunoblotting. â-actin served as an internal control. 4. (**B**) Nef expressed by HIV-1 induces release of IL-6. The immDCs were infected with either wt, *Änef*, *Äenv*, *Änef*/*Äenv* (VSV-G) HIV-1. Cells were extensively washed and re-fed after infection. Supernatants were collected at 12 hrs and 24 hrs of infection. The content of IL-6 was then measured by ELISA. Data represent the mean±SEM (n = 3). Statistical analysis was performed using Student's t-test, with the levels of significance defined as p*<0.05 and p**<0.01. 4. (**C**) Flow cytometry analysis for the detection of infection in immDCs. After 24 hrs of infection with either wt, *Δenv*, *Δnef* or *Δenv*/*Δnef* were analysed for the detection of the HIV-1 antigen (p24) by incubating with anti-p24 HIV-1 monoclonal antibody followed by staining with PE-conjugated anti mouse antibody. PE-conjugated isotype matched IgG served as control. Presence of histogram peaks evidenced the occurrence of infection and validated our results.

Several cytokines have been reported to be produced by both envelope glycoprotein gp120 [27]–[30] and Nef [31], [32]. Hence we studied the events that occur when gp120 and Nef act in the presence of each other. As a first step we analysed the effect of HIV-1 gp120 on Nef mediated cytokine release on immDCs. The cells were subjected to treatment with gp120 and Nef separately and also combining the both. The cytokines MIP-1alpha, MIP-1beta, IL-6, IL-10 and TNF-alpha were ascertained in the presence of gp120, Nef and combination of gp120 and Nef, respectively (Fig. 1). Interestingly, it was observed that the level of IL-6, which was induced by Nef, dropped significantly on subjecting to treatment with both gp120 and Nef in immDCs. The experiment was repeated with Nef and heat denatured gp120 proteins in immDCs (Fig. 2), which showed that active gp120 interacts with Nef and causes a downregulatory effect on Nef induced IL-6 release. We next performed experiments with lower concentration of HIV-1 proteins to see whether the effect of gp120 on Nef could be achieved at lower doses (in the context of HIV-1 infection). For this, we challenged the immDCs with Nef (Fig. 3A and Fig. 3B) and gp120 (Fig. 3C and Fig. 3D) proteins separately in independent sets of experiments under similar conditions to observe the effect in both dose and time dependent manner. The results indicated that the maximum release of cytokines from immDCs occurred at concentrations of 4 µg/ml for gp120 and 100 g/ml for Nef. The downregulatory effect of gp120 on Nef was then checked with infectious molecular clones. For this we infected the immDCs with 1 ng/10^5^ cells of (VSV-G) pseudotyped HIV-1 Δ*nef*, HIV-1 Δ*env* and HIV-1 Δ*env*/Δ*nef* strains along with wild type ones. The expression of Nef was first verified in the immDCs infected with the aforesaid viruses (Fig. 4A). The supernatants from immDCs were collected at 12 and 24 hrs postinfection and the level of IL-6 was measured through ELISA. The concentration obtained was much less compared to those cells treated with recombinant Nef and gp120 proteins. However the IL-6 suppression level could be easily distinguished in intact *env* and *env* defective pseudoviruses (Fig. 4B). The infection of immDCs by aforesaid HIV-1 viruses was evaluated through flow cytometry (Fig. 4C). The rest of the results were verified with molecular clones of HIV-1 (data not shown).

### Downregulation of Nef dependent IL-6 release relies on the interaction of gp120 with DC-SIGN

**Figure 5:**
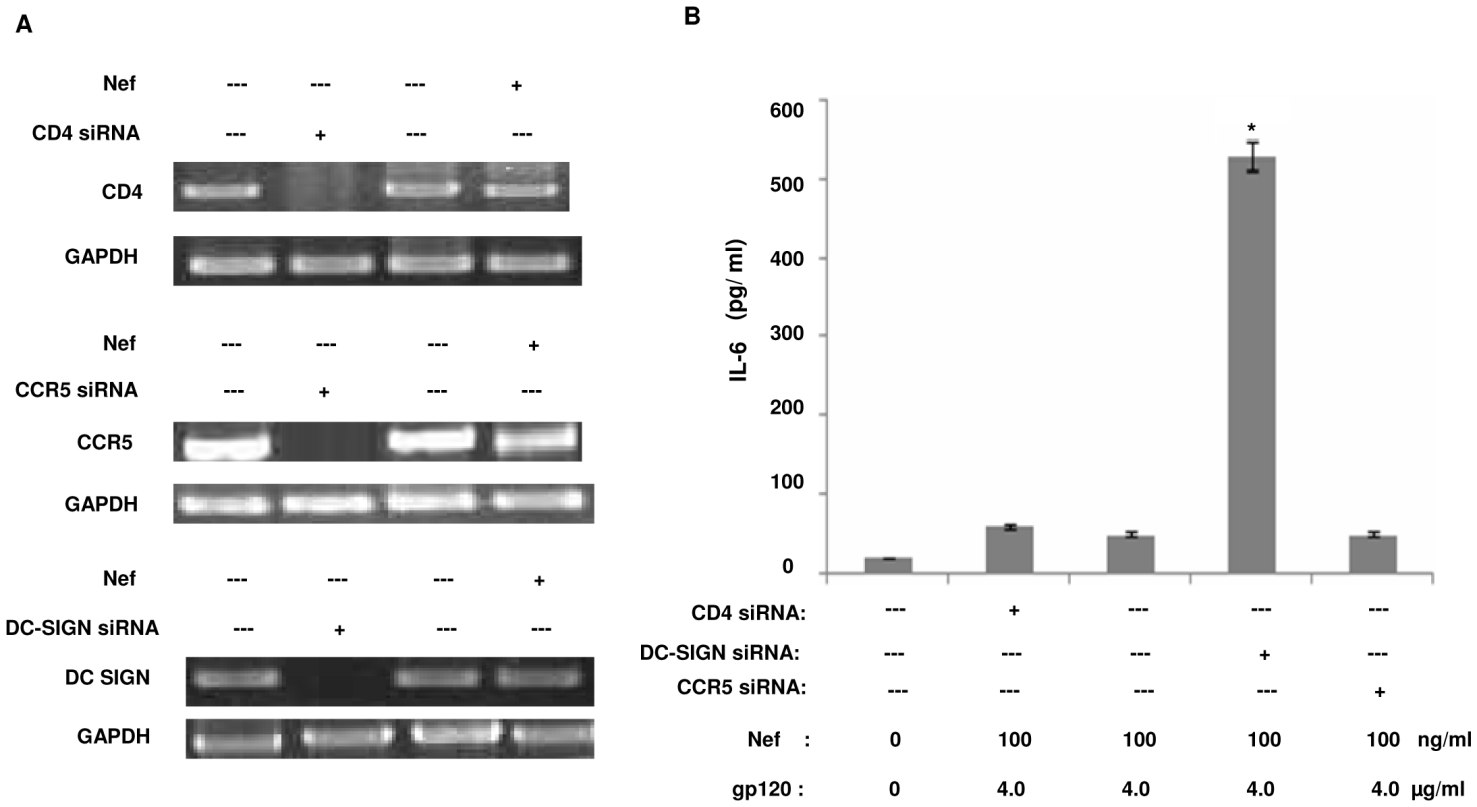
Effect of receptor silencing on IL-6 concentration in the presence of Nef and gp120. (**A**) Efficacy of CD4, CCR5 and DC-SIGN silencing mediated through specific siRNA (5 nM/ml) transfections in immDCs. The expression of specific receptors in immDCs was analysed both in the presence and absence of Nef. The specific genes were checked using appropriate primers through RT-PCR. GAPDH served as internal control (**B**) ELISA of IL-6 concentration in pg/ml in immDCs in the presence and absence of CD4, CCR5 and DC-SIGN. X-axis represents the presence or absence of CD4 siRNA, DC-SIGN siRNA and CCR5 siRNA by ‘+’ and ‘---’ signs respectively in immDCs in the presence of both Nef and gp120. The concentration of siRNA used was 100 nM. Y-axis represents the concentration of IL-6 in pg/ml. Data represent the mean±SEM (n = 3). Statistical analysis was performed using Student's t-test, with the levels of significance defined as p*<0.05.

Although the engagement of CD4 and other chemokine receptors by gp120 is required for viral infection [6], [33], yet data have shown the involvement of C-type lectins such as DC-SIGN in HIV-1 recognition by binding the HIV-1 gp120 [9]. Since gp120 was found to diminish Nef induced IL-6, hence we wanted to find out whether any receptor interaction by gp120 was involved in the inhibition of IL-6. The primary receptors present on the surface of immDCs, i.e. CD4, CCR5 and DC-SIGN were targeted with which gp120 generally bind. The expression of these receptors in immDCs were analysed through FACS (data not shown) and the receptors were silenced through specific siRNA individually. The efficacy of receptor silencing both in the presence and absence of Nef protein has been shown in Fig. 5A.

It was noted that silencing of CD4 and CCR5 did not cause any alteration to the effect of gp120 on IL-6. The diminishing of IL-6 by gp120 was observable despite knocking down both CD4 and CCR5. However, DC-SIGN silencing hindered the effect of gp120. The cells transfected with DC-SIGN siRNA showed enhanced expression of IL-6 compared to those where the receptor was present (Fig. 5B). This proved that gp120 needs to interact with DC-SIGN in order to carry out its inhibitory effect on IL-6. Our observation was further confirmed by RT-PCR (data not shown), where similar results were obtained. This established that IL-6 release induced by Nef protein is downregulated by gp120 via DC-SIGN receptor.

### Association of gp120 with DC-SIGN, releases IL-10 which mediates the downregulation of Nef dependent IL-6 release

**Figure 6:**
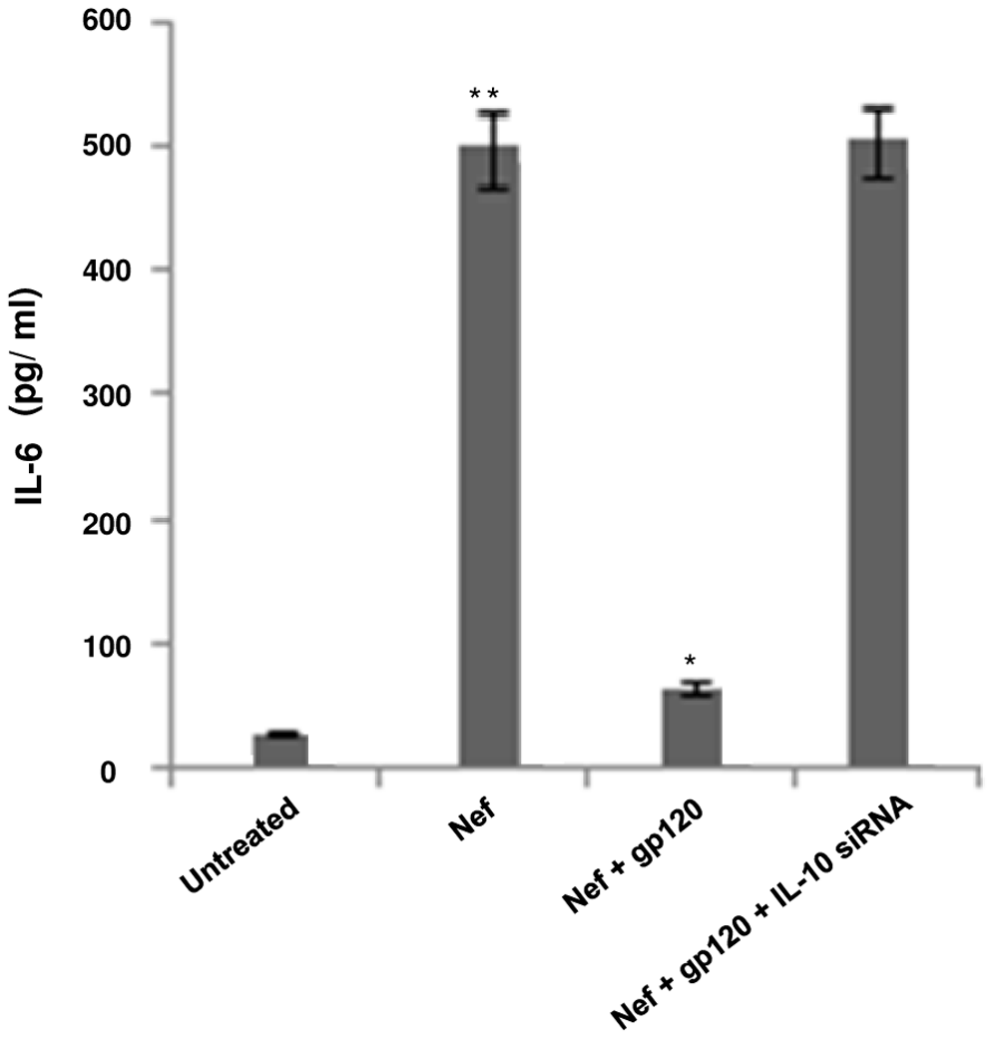
IL-10 knock down inhibits the downregulatory effect of gp120 on Nef mediated IL-6 expression. ELISA of IL-6 concentration in pg/ml was performed in immDCs treated with Nef, combination of Nef and gp120 and in the presence of IL-10 siRNA. Data represent the mean±SEM (n = 3). Statistical analysis was performed using Student's t-test, with the levels of significance defined as p*<0.05 and p**<0.01.

It has been evidenced that gp120 can interact with DC-SIGN to induce IL-10 release [34]. However, it is not known whether this IL-10 can exert downregulation of IL-6 induced by Nef. Hence we verified the role of IL-10 by transfecting immDCs with IL-10 specific siRNA followed by confirming IL-6 expression. The results showed that knock down of IL-10 in immDCs failed to exhibit the inhibitory effect of gp120 on Nef induced IL-6 (Fig. 6), i.e. the level of IL-6 expression in the presence of IL-10 siRNA transfected cells was similar to the ones treated with Nef protein alone in the absence of gp120. In this regard, it should be mentioned that gp120 can induce IL-10 release in cell lines, both in the presence and the absence of DC-SIGN. However, gp120 can downregulate the expression of IL-6 only when it interacts with DC-SIGN, perhaps because the augmented release of IL-10 by gp120 on binding with DC-SIGN mediates the inhibitory effect of gp120.

### The IL-10 release by gp120 depends on the activation of Raf-1 along with NF-kappa beta

**Figure 7:**
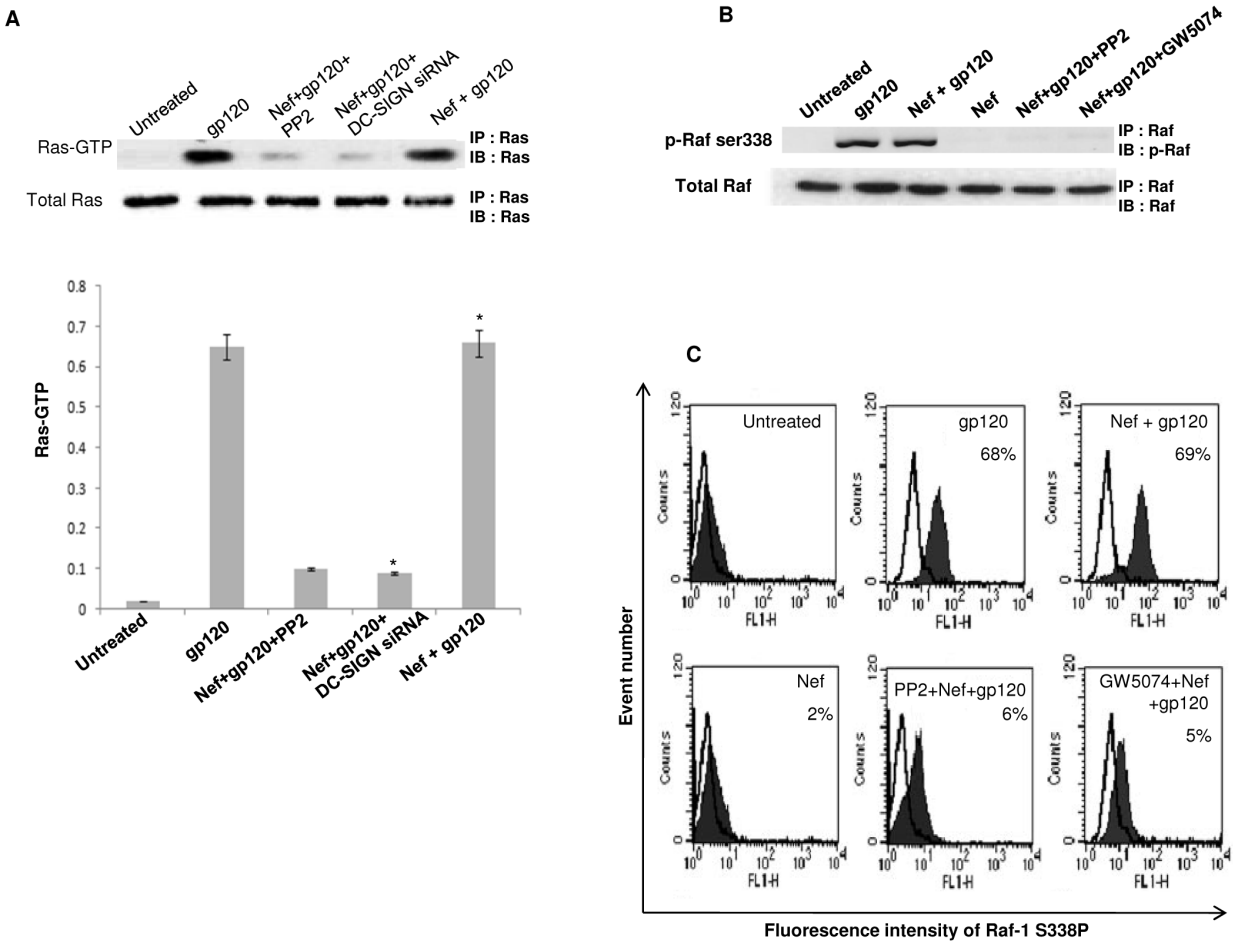
Ras-GTP and Raf-1 activation is required for gp120 to implement its downregulatory effect on Nef induced IL-6. (**A**) Immunoblot analysis for the detection of Ras-GTP in immDCs. Ras-GTP activation was measured in presence of gp120; Nef, gp120 and kinase inhibitor (PP2); Nef, gp120 and DC-SIGN siRNA; Nef and gp120. The upper panel of the western blot analysis reveals that Ras-GTP gets activated in the presence of gp120, both in the presence (5^th^ lane) and absence (2^nd^ lane) of Nef. This shows that Nef has no role in the activation process. However, presence of kinase inhibitor (3^rd^ lane) and knock down of DC-SIGN (4^th^ lane) inhibit the activation of Ras-GTP. The lower panel of the blot denotes the total Ras in the cells. The densitometric analysis corresponds to the respective bands obtained in immunoblotting. Data represent the mean±SEM (n = 3). Statistical analysis was performed using Student's t-test, with the levels of significance defined as p*<0.05. (**B**) Western blot analysis of Raf-1 phosphorylation at ser338 residue in presence of gp120; Nef and gp120; Nef; Nef, gp120 and kinase inhibitor (PP2); Nef, gp120 and GW5074. The upper panel of the blot clearly shows that Raf-1 phosphorylation is facilitated by gp120. The lower panel denotes the total Raf-1 content of the cell. Data represent the mean±SEM (n = 3). (**C**) FACS analysis of Raf-1 phosphorylation at ser338 residue in presence of gp120; Nef and gp120; Nef; Nef, gp120 and kinase inhibitor (PP2); Nef, gp120 and GW5074 (Raf-1 inhibitor). The percentage on the upper right hand side of each histogram denotes the cell percentage positively stained.

**Figure 8:**
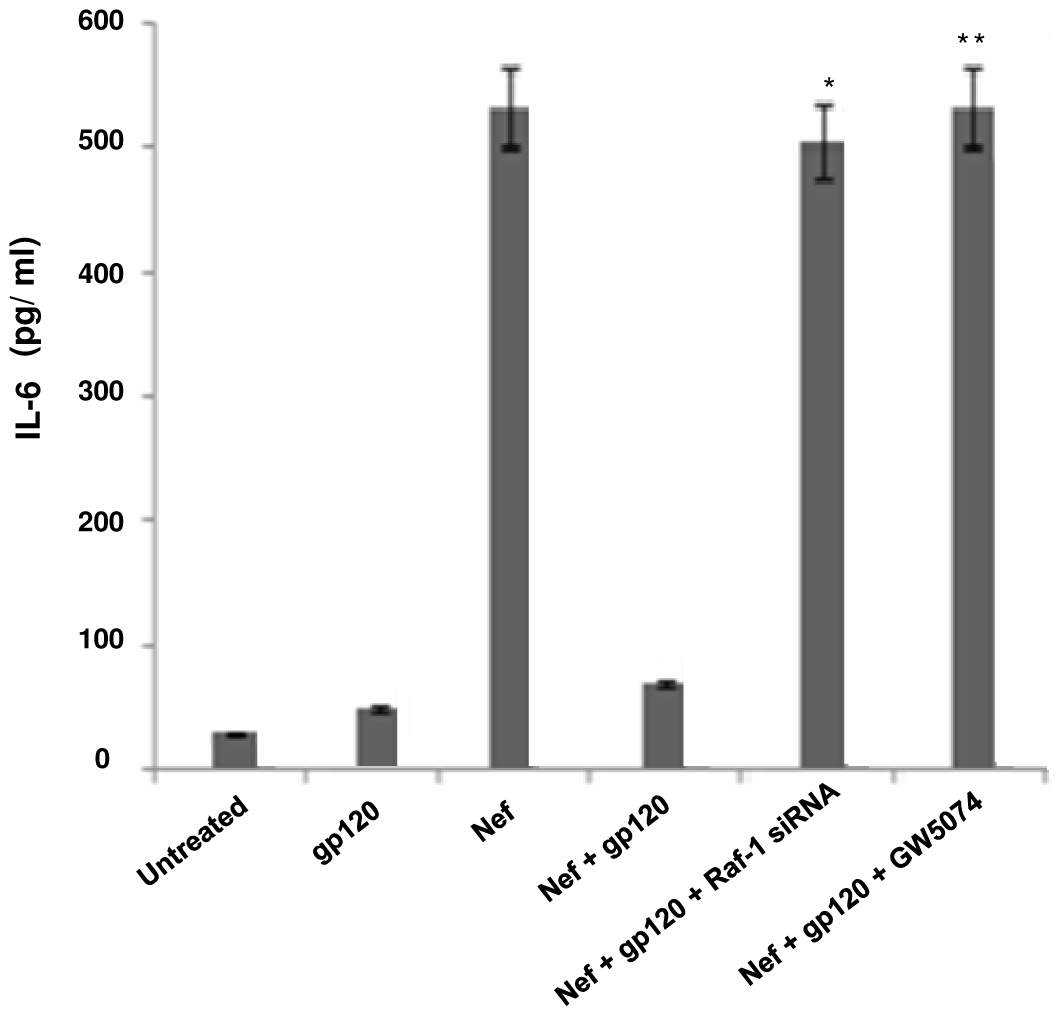
Inhibition of Raf-1 prevents the downregulatory effect of gp120 on Nef induced IL-6. ELISA of IL-6 concentration in pg/ml was performed in immDCs in presence of gp120; Nef; Nef and gp120; Nef, gp120 and Raf-1 siRNA; Nef, gp120 and GW5074. Data represent the mean±SEM (n = 3). Statistical analysis was performed using Student's t-test, with the levels of significance defined as p*<0.05, p**<0.01.

**Figure 9:**
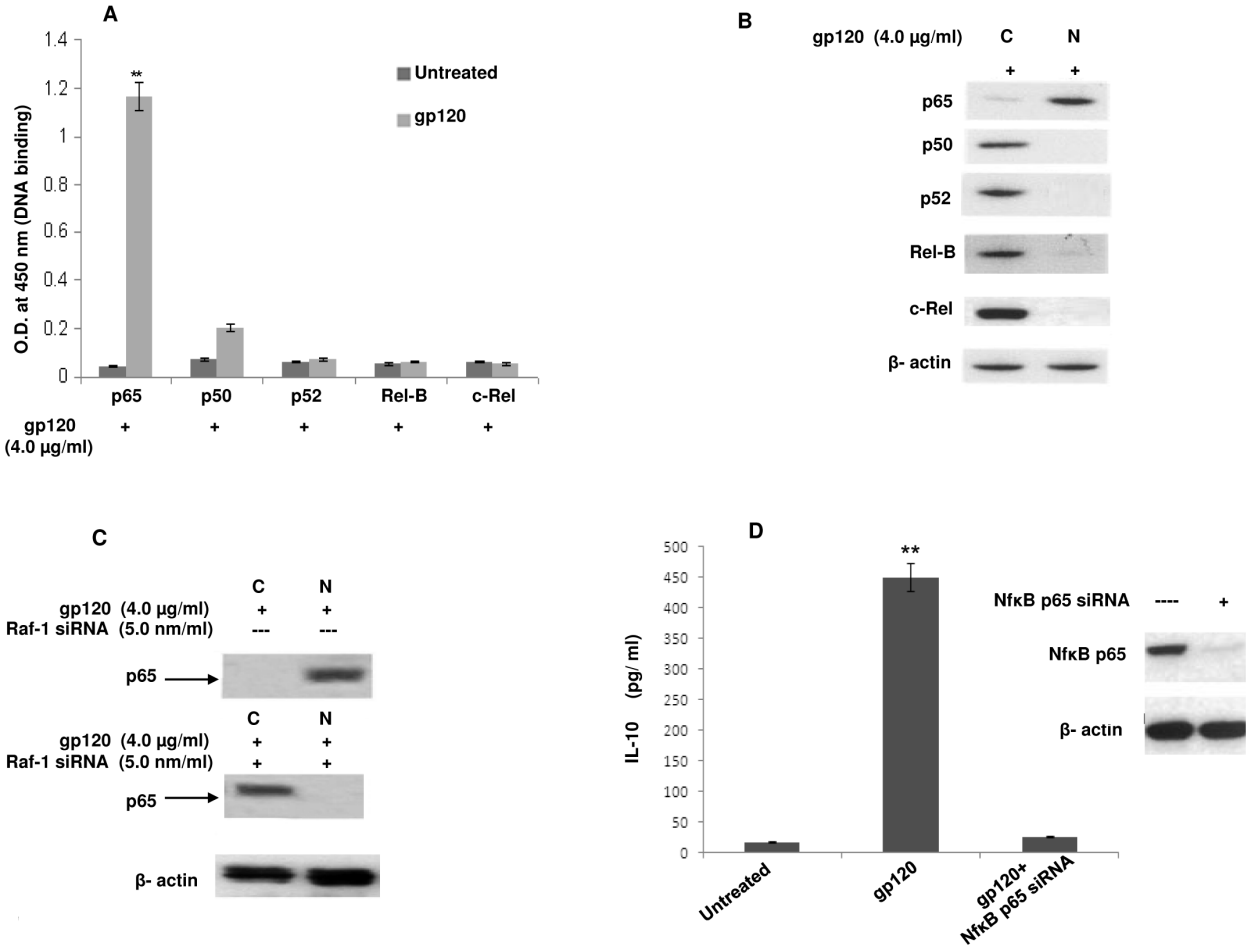
Translocation of different NF-êB subunits (p65, p50, p52, Rel-B, c-Rel) in immDCs from cytosol denoted as ‘C’ to nucleus denoted as ‘N’ in presence of gp120 (4.0 µg/ml). (**A**) DNA- binding ELISA analysis of the different subunits of NF-êB in the presence of gp120. Data represent the mean±SEM (n = 3). Statistical analysis was performed using Student's t-test, with the levels of significance defined as p*<0.05, p**<0.01. (**B**) Immunoblotting analysis of the translocation of the different subunits of NF-êB from cytosol denoted as ‘C’ to nucleus denoted as ‘N’. â- actin served as the internal control. (**C**) Immunoblot analysis of the translocation of p65 subunit of NF-êB from cytosol denoted as ‘C’ to nucleus denoted as ‘N’ in presence of gp120 and a combination of gp120 and Raf-1 siRNA. â- actin served as the internal control. (**D**) Effect of NF-êB p65 silencing in the induction of gp120 dependent IL-10 release. The immunoblot denotes the efficacy of silencing p65 subunit with â-actin serving as internal control. The bar diagram denotes the diminished expression of IL-10 in the presence of NF-êB p65 siRNA. Data represent the mean±SEM (n = 3). Statistical analysis was performed using Student's t-test, with the levels of significance defined as p**<0.01.

The involvement of DC-SIGN with gp120 encouraged us to trace the downstream signaling of DC-SIGN in order to verify whether IL-10 release relies on the activation of any other signaling molecule that gets induced after gp120-DC-SIGN interaction. It has been evidenced that HIV-1 can mediate the activation of Raf-1 kinase through the DC-SIGN signalosome, thus modulating the cytokine responses to HIV-1 [35]. Raf-1 activation occurs through an interaction with the active form of Ras-GTPase [36]. Also, Raf-1 activation by DC-SIGN can induce phosphorylation of NF-êB to shape the adaptive immunity [35]. Hence, Ras-GTPase assay was performed to analyse DC-SIGN dependent Raf-1 triggering by gp120. The immunoblotting experiments showed that incubating immDCs with gp120 led to the activation of Ras-GTP, which remained unaffected in presence of Nef protein. Further, silencing of DC-SIGN receptor through specific siRNA and kinase inhibitor (PP2) in separate experiments blocked the Ras-GTP activation mediated by gp120 (Fig. 7A). Next we checked the involvement of Raf-1, one of the downstream effector molecule of Ras pathway. It was observed that gp120 could not mediate the phosphorylation of Raf-1 in immDCs by pre treating the cells with PP2 (kinase inhibitor) and GW5074 (Raf-1 inhibitor) separately in independent experiments through immunoblotting and FACS (Fig. 7B and Fig. 7C). This indicated that gp120 leads to the activation of Ras followed by Raf in immDCs by triggering DC-SIGN receptors. The knock down of Raf-1 by Raf-1 specific siRNA and also treatment with Raf-1 inhibitor GW5074 in immDCs, in separate sets of experiments, reversed the diminishing effects generated by gp120 on Nef induced IL-6 expression i.e. gp120 could not downregulate Nef induced IL-6 in presence of Raf-1 siRNA and GW5074 (Fig. 8). Furthermore, it was also seen that incubation of immDCs with gp120 causes activation of NF-êB, as evidenced by the ELISA and immunoblotting experiments performed for the investigation of p65, p50, p52, Rel-B and c-Rel subunits of NF-êB from the cytosol and nuclear extracts of immDCs. Our experiments revealed that p65 subunit is activated by gp120 (Fig. 9A) which then gets translocated to nucleus from cytosol (Fig. 9B). Silencing of Raf-1 through specific siRNA prevents the translocation of p65 subunit of NF-êB from cytosol to nucleus induced by gp120 (Fig. 9C), which proved that NF-êB activation by gp120 relies on Raf-1 phosphorylation. Interestingly, it was also evidenced that silencing of NF-êB diminished the IL-10 induction augmented by gp120 (Fig. 9D). This proved that the activation of both Raf-1 and NF-êB downstream DC-SIGN are necessary in order to facilitate the downregulation of IL-6 by gp120.

### The activation of STAT3 by Nef induced IL-6 gets suppressed on activation of IL-10 through gp120

**Figure 10:**
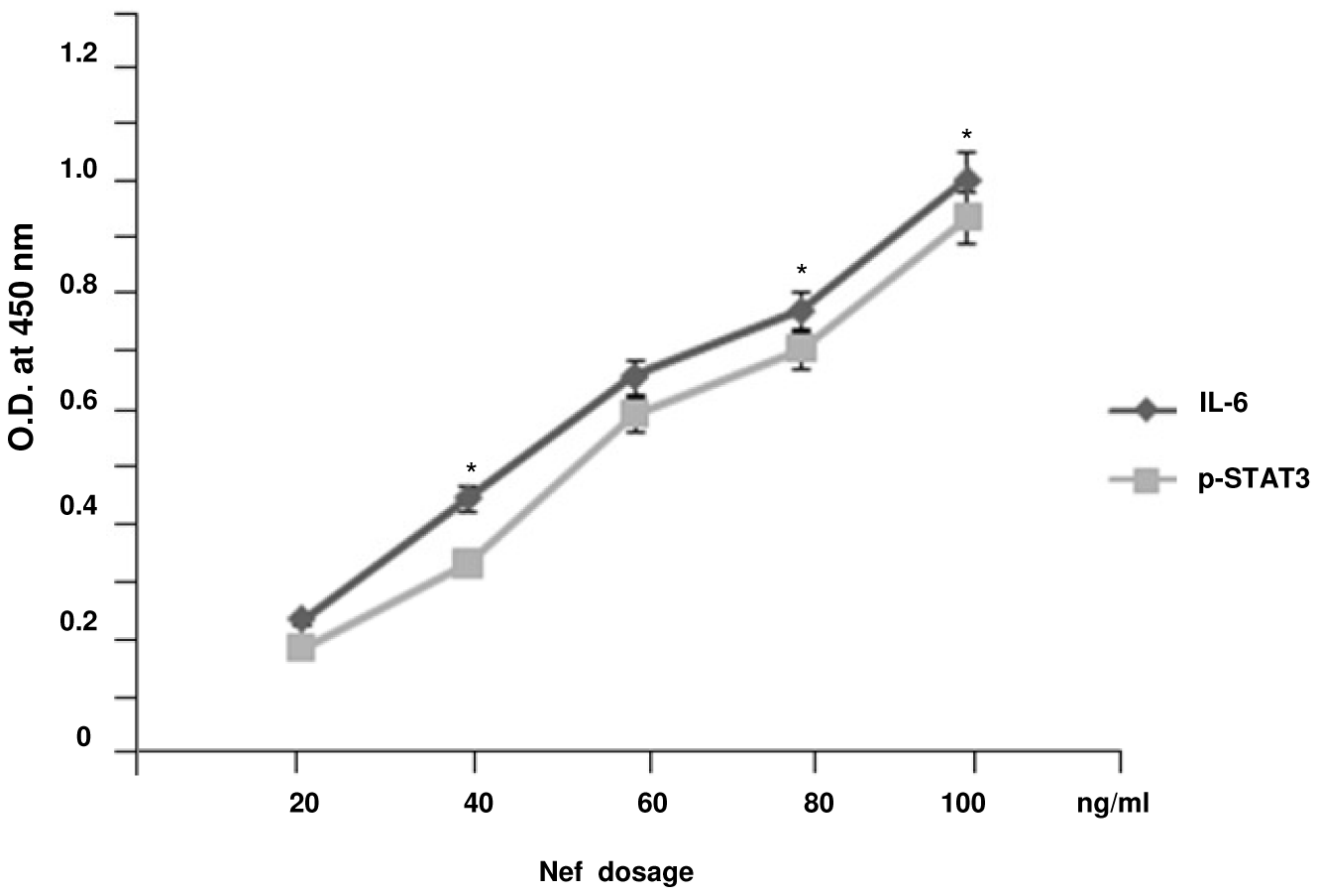
Nef induces phosphorylation of STAT3 along with IL-6 in a dose-dependent manner. ELISA analysis showed that both IL-6 and p-STAT3 get induced by Nef in immDCs. The X- axis denotes the dosage of Nef in ng/ml and the Y-axis denotes the OD obtained at 450 nm. Data represent the mean±SEM (n = 3). Statistical analysis was performed using Student's t-test, with the levels of significance defined as p*<0.05.

**Figure 11:**
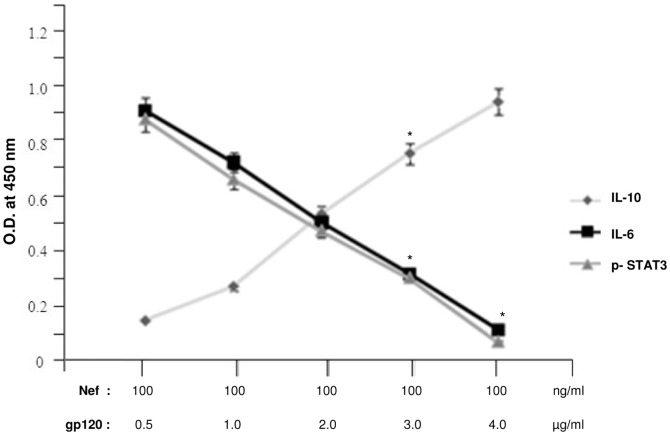
gp120 activates IL-10 in a dose dependent manner unlike IL-6 and IL-6 induced STAT3 phosphorylation which get downregulated. ELISA analysis showed the increase of IL-10 expression through gp120 in contrast to IL-6 and IL-6 induced STAT3 phosphorylation which exhibited a gradual decrease in concentration depending on the dosage of gp120. The X-axis shows the combined dosage of Nef and gp120, where Nef dosage has been kept fixed at 100 ng/ml and the gp120 dosage has been varied from 0.5 µg/ml to 4.0 µg/ml. The Y-axis denotes the OD obtained at 450 nm. Data represent the mean±SEM (n = 3). Statistical analysis was performed using Student's t-test, with the levels of significance defined as p*<0.05.

STAT proteins are the effectors of cytokine signaling in several hematopoietic cell lineages [37], [38], some of which are targets for HIV-1 infection. STAT3 activation is thought to have a role in cell survival, as seen by the resistance to the anti- apoptotic effect of IL-6 in STAT3 defective T lymphocytes [39]. The effect of Nef induced IL-6 in immDCs was verified to find out whether IL-6 release in response to activation by Nef, is able to induce STAT3. Our experiment revealed that Nef induces phosphorylation of STAT3 along with the induction of IL-6 in a dose dependent manner (Fig. 10). Silencing IL-6 in immDCs with IL-6 specific siRNA failed to cause STAT3 activation, thus indicating the involvement of IL-6 in Nef dependent STAT3 activation. Our study elucidated that in the presence of gp120, Nef dependent STAT3 activation gets suppressed (Fig. 11). This indicated that gp120 inhibits STAT3 activation by interrupting Nef induced IL-6 secretion via IL-10 release.

### Gp120 recruits SOCS-3 through IL-10 activation for suppressing IL-6 and IL-6 dependent STAT3

**Figure 12:**
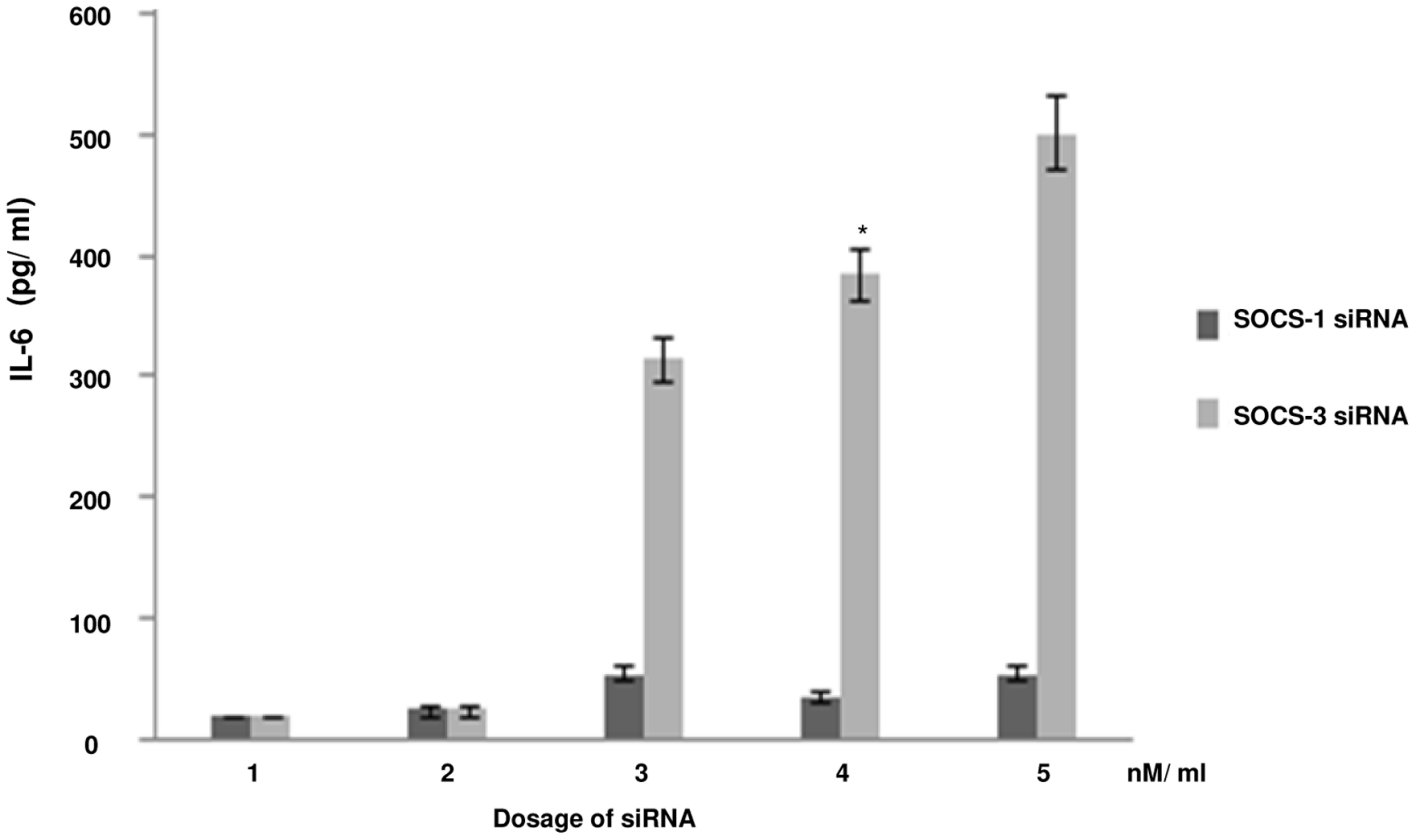
IL-6 expression in immDCs on silencing SOCS-1 and SOCS-3. ELISA analysis reavealed the diminishing of IL-6 expression on subjecting immDCs, incubated with Nef and gp120, to SOCS-1 knock down. However, silencing SOCS-3 caused an increase in the expression of IL-6 in a dose dependent manner. The X-axis denotes the dosage of SOCS-1 siRNA and SOCS-3 siRNA in nM/ml. The Y-axis denotes the concentration of IL-6 in pg/ml. Data represent the mean±SEM (n = 3). Statistical analysis was performed using Student's t-test, with the levels of significance defined as p*<0.05.

**Figure 13:**
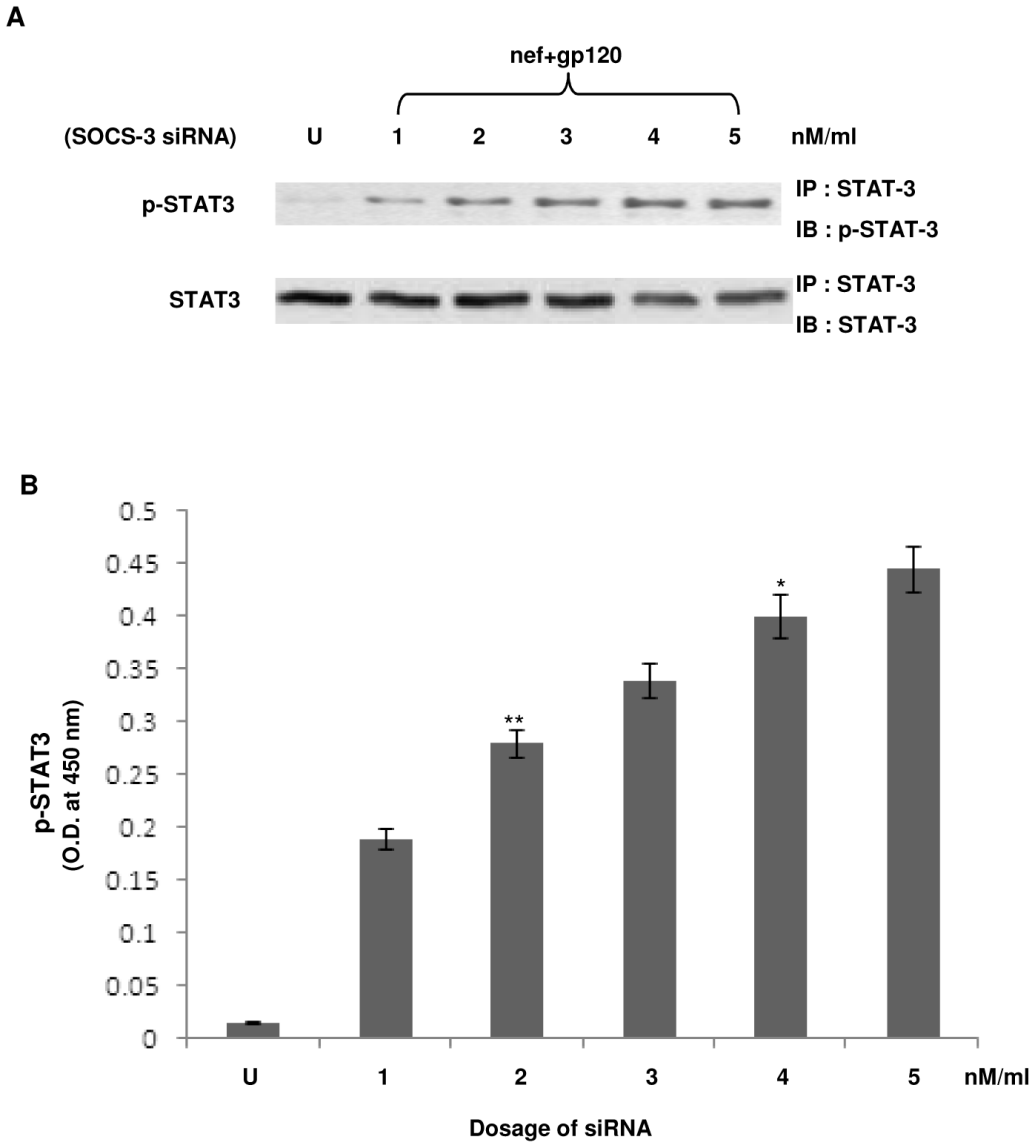
Effect of SOCS-3 silencing on STAT3 phosphorylation in the presence of Nef and gp120. (**A**) Western blot analysis denotes the phosphorylation status of STAT3 on subjecting immDCs incubated with Nef+gp120 to SOCS-3 siRNA in a dose dependent manner. The upper panel refers to p-STAT3 whereas the lower panel denotes the total STAT3 in immDCs. ‘U’ refers to untreated cells and 1–5 denote the dosage of SOCS-3 siRNA in nM/ml. (**B**) ELISA analysis of p-STAT3 under the same conditions. The X-axis denotes the dosage of SOCS-3 siRNA and the Y-axis denotes the OD obtained at 450 nm. Data represent the mean±SEM (n = 3). Statistical analysis was performed using Student's t-test, with the levels of significance defined as p*<0.05, p**<0.01.

It is known that cytokine stimulation induces a family of SOCS proteins, whose predominant function is to block the generation of the STAT signal from a cytokine receptor [40]. Genetic evidence from mice and cells lacking SOCS-1 and SOCS-3 have showed that these two proteins are required to reduce overall signaling output from their target receptors [40]. Our experiments revealed that IL-10 secreted by the interaction of gp120 with DC-SIGN was responsible for diminishing IL-6 release. However, we wanted to verify whether IL-10 was the terminal downstream factor of gp120 mediated DC-SIGN signaling involved in diminishing IL-6 release or, whether the effect was being mediated through the interplay of any cytokine induced inhibitor. Since SOCS-1 and SOCS-3 are reported to be activated through IL-10 [41], hence we performed the knock down of both SOCS-1 and SOCS-3 in immDCs separately before proceeding further. The efficacy of SOCS-1 and SOCS-3 knock down was confirmed by RT-PCR (data not shown). It was seen that SOCS-1 silencing did not create any alteration to the effect of IL-6 down regulation by gp120 activated IL-10, but transfection of the cells with SOCS-3 siRNA reversed the obtained results (Fig. 12). The results clearly indicated that the effect of gp120 on Nef was neutralized in SOCS-3 silenced immDCs, i.e., gp120 mediated IL-10 release could not exhibit suppression of Nef dependent IL-6 release in absence of SOCS-3. This signified the role of SOCS-3 in this signaling cascade, since it emphasized on the fact that IL-10 can bring about the downregulation of IL-6 only through the involvement of SOCS-3. Further SOCS-3 silencing revealed that gp120 was unable to suppress STAT3 activation induced by Nef (Fig. 13).

### Gp120 induces apoptosis by suppressing STAT3 induced by Nef

**Figure 14:**
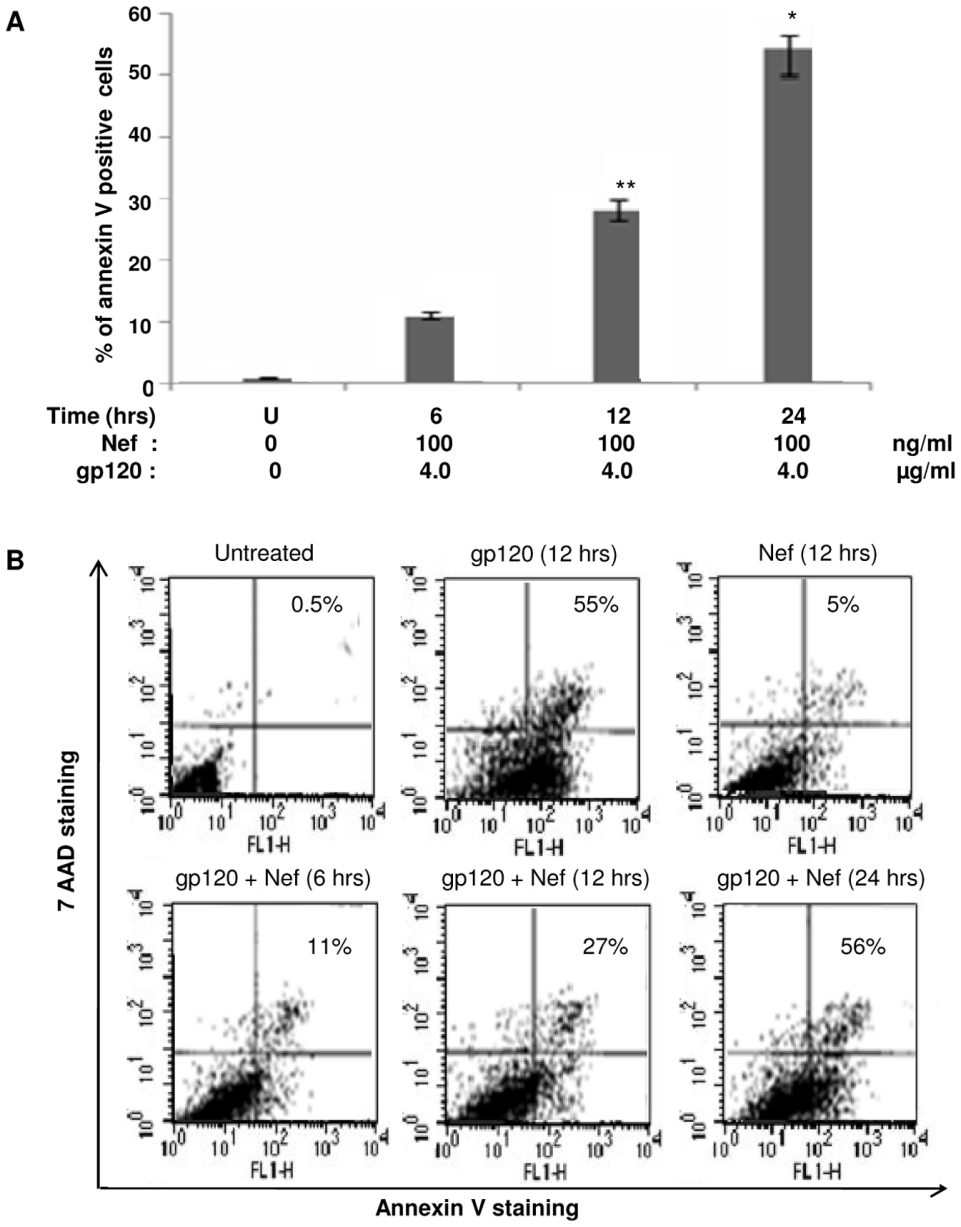
Relative measurement of the rate of apoptosis in immDCs upon subjecting them to treatment with Nef and gp120. (**A**) ELISA analysis of the apoptosis rate in immDCs upon subjecting them to treatment with Nef and gp120 in individual sets of experiments. The dosage of Nef and gp120 were 100 ng/ml and 4 µg/ml respectively. X-axis denotes the combined dosage of Nef and gp120. Y-axis represents the percentage of annexin positive cells. Data represent the mean±SEM (n = 3). Statistical analysis was performed using Student's t-test, with the levels of significance defined as p*<0.05 and p**<0.01. (**B**) FACS analysis of apoptotic immDCs cells on treating with gp120, Nef and combination of gp120 and Nef in a time dependent manner. The percentage denoted on the right side of each histogram denotes the positively stained cells.

Several evidences suggest the role of HIV-1 in inducing apoptosis in many cell lines [42], [43]. However, treating immDCs with Nef did not exhibit any such event, thus showing that Nef induces anti-apoptotic signal via activation of STAT3. Upon incubating Nef pretreated immDCs with gp120, it was observed that the anti-apoptotic effect of STAT3 gets diminished in a time dependent manner (Fig. 14). This brings out the fact that gp120 suppresses the induction of STAT3 by Nef, which ultimately reverses the anti-apoptotic signals, thus leading to complete apoptosis.

## Discussion

AIDS pathogenesis relies on the interplay of molecular mechanisms that determine the immunological perspective. In the present study we have shown an *in vitro* cross talk between gp120 and Nef, which individually stand out as important HIV-1 proteins responsible for the viral pathogenesis. Nef induces IL-6 response in immDCs, which has numerous effects including the activation of STAT3. The STAT family includes seven elements [37], [38] whose activation is involved in the response of a wide number of cytokines, growth factors, and hormones. The promoters of numerous anti-apoptotic genes are stimulated by activated STAT3 and it has been witnessed that the expression of Nef counteracts the HIV-1 induced apoptosis in lymphocytes [44]–[46]. It can also be said that the cell survival mechanism is generated by STAT3 activation, as reported by the resistance to the antiapoptotic effect of IL-6 in STAT3-defective T lymphocytes [39]. Thus STAT3 activation in the Nef-expressing cells is important for the interpretation of the role played by the Nef protein in AIDS pathogenesis. Our results have demonstrated that the effect of Nef is nullified in immDCs through IL-10. gp120 induces IL-10 release which in a time dependent manner causes gradual inhibition of the anti-apoptotic effect of Nef by suppressing the activation of STAT3. In other words, Nef incubated immDCs cells, when treated with gp120, exhibits apoptosis. The IL-10 release by gp120 is not a direct mechanism and depends on the involvement of other factors such as Ras, Raf and NF-êB activation. The IL-10 then binds to the IL-10 receptors present on the surface of immDCs which causes the activation of SOCS-3. The downregulatory effect of gp120 on Nef is then carried out by SOCS-3 which actually blocks the IL-6 induction by Nef.

**Figure 15:**
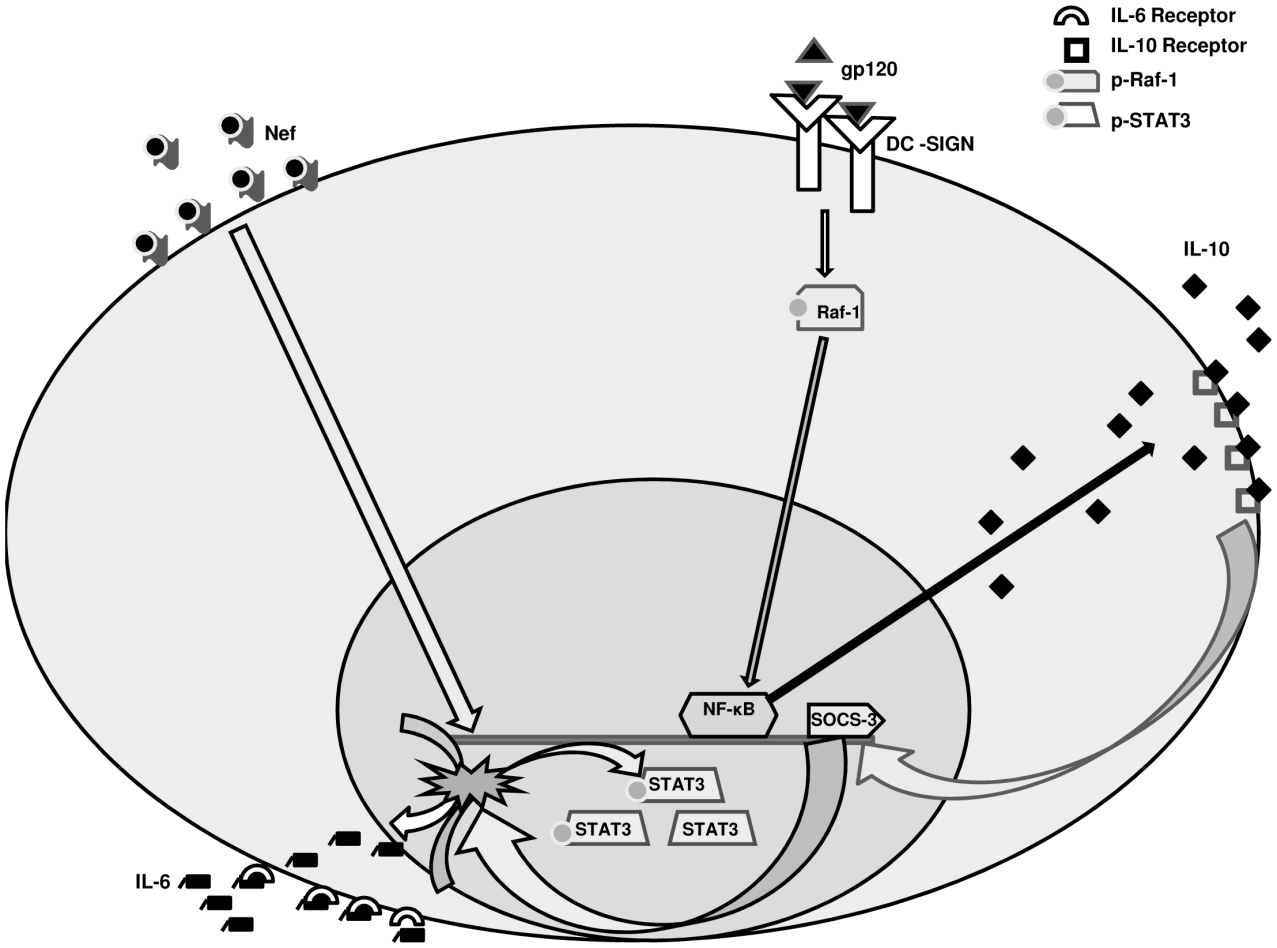
Schematic diagram of gp120 mediated downregulation of Nef induced IL-6 and STAT3 phosphorylation in immDC.

Though gp120 can interact with different chemokine receptors to carry out viral infection, yet it was evident from our observations that the binding of gp120 to DC-SIGN receptor is crucial for the downregulation of Nef by gp120. In this regard it is important to mention that gp120 has various effects on DCs including generation of immunosuppression [34]. The DC-SIGN receptor present on the surface of immDCs interacts with HIV-1 gp120 and leads to the activation of Ras-GTPase activity which further activates Raf-1. We have shown that the downregulatory effect of gp120 on Nef involves the cooperation of several signal molecules which are interlinked and finally lead to the obtained result. gp120 interacts with DC-SIGN to induce Raf-1 by activating Ras-GTPase. This further leads to the activation of NF-êB which is evident from translocation of the p65 fragment of NF-êB in the nucleus and thereby triggers IL-10 release. IL-10 in turn causes SOCS-3 activation which ultimately delivers the downregulatory effect of gp120 on Nef induced IL-6 and STAT3 in immDCs. The putative illustration of the entire study has been represented in Fig. 15. The study of the influences of gp120 appears to be of profound interest since it interacts with the host receptors primarily during the viral life cycle. In this study, we have analysed that in immDCs, the interaction of gp120 with other host receptors (CD4, CCR5) does not put any impact on Nef induced cytokines (IL-6, TNF-alpha etc). However, gp120- DC-SIGN interaction leads to the downregulation of IL-6 expression. Since Nef mediated anti-apoptotic effects are crucial for viral replication in the host, hence there is every probability that after accomplishing viral replication, HIV-1 switches off the anti- apoptotic effect of Nef by suppressing the STAT3 activation in immDCs.

It is still unclear whether the DC-SIGN expressing immDCs are kept alive by the virus through activation of anti-apoptotic genes until it reaches the lymph node and then sacrificed to carry on infection. However, the significance of the cross-talk between the signaling pathways of Nef and gp120 cannot be over looked, since it could make a better understanding of the process by which HIV-1 modulates signaling in immDCs. This could be vital in the development of new therapeutic approaches that might ultimately restrict or decrease the size of cellular virion reservoirs in HIV-1-infected patients.

## Materials and Methods

### Cell culture and cell treatment

Human monocytes were isolated from buffy coats of human blood (the approval was provided by Institutional Ethical Committee of National Institute of Cholera and Enteric Diseases) with a Ficoll gradient, followed by hypertonic density centrifugation in Percoll. After 30-mins culture in RPMI supplemented with 5% human serum and 1% L-glutamine, the monocytes became adherent and were washed thrice with SMEM Spinner medium to remove contaminating lymphocytes.

In differentiation experiments, monocytes were seeded at 5×10^5^ cells/ml in tissue culture dishes with no change of the culture medium after addition of the differentiation inducer(s). Unless otherwise indicated, IL-4 was added into the cells 24 hours after treatment with GM-CSF (15 ng/ml) (Calbiochem, USA) and differentiation allowed to proceed for 72 hours. For subsequent analysis, differentiated monocytes were detached from tissue culture plates by incubation in PBS on ice.

The immDCs obtained from monocytes were used for experimental purpose on the 3rd day of their differentiation. The cells were treated with recombinant Nef (upto 100 ng/ml) and gp120 (upto 4 µg/ml) protein. The gp120 protein was purified from HIV-1 CCR5 isolate BaL in monomeric form [47] (AIDS Research and Reference Reagent Program of the Division of AIDS, National Institute of Allergy and Infectious Diseases, US National Institutes of Health). Each sample was processed after addition of 1 volume of 1/2 PBS, using the Endofree Red 5/1 Endotoxin removal kit (Profos AG). After endotoxin removal the endotoxin levels were <3 EU/mg.

Immature DCs were separately treated with PP2 (20 mM) (Calbiochem, USA), GW5074 (25 mM) (Calbiochem, USA), BAY-11-7082 (20 mM) (Calbiochem, USA) in different sets of experiments.

### Virus preparation and infection

The NL4-3 HIV-1 was prepared following the protocols as described elsewhere [48] and pseudotyped with the envelope glycoprotein of the Vesicular Stomatites Virus (VSVG). These viruses were obtained as supernatants of 293 T cells after 48 hrs. This was mediated by calcium phosphate co- transfection of the pUc19/NL4-3 molecular clone in a molar ratio of 5∶1 with a plasmid expressing the VSV-G with early CMV promoter. Ultracentrifugation was performed to clarify and concentrate supernatant [49]. Briefly, the Ä*env* HIV-1 molecular clone was constructed by inserting the NL4-3 SalI/BamHI fragment deleted of the BglII/BglII region in the SalI and BamHI sites of a pUc19/NL4-3 derivative lacking the whole *nef* gene and carrying a polylinker at the end of the *env* gene. The HIV-1 capsid (CA) p24 contents were measured by quantitative ELISA (Perkin Elmer, USA). A total of 10 ng of pseudotyped HIV-1 was used to infect immDCs. The experiment of viral adsorption was carried out in 24-wells plates followed by incubation of cells for 1 h at 37°C with the viral inoculum (dilution performed in 100 µl of complete medium). Later, after the removal of viral inoculum, cells were washed and supplemented with 300 µl of complete medium. FACS analysis was performed to calculate the percentage of cells expressing intracytoplasmic HIV-1 p24 related products. The cells were at first incubated with FACS permeabilization buffer (Santacrutz, USA). The primary labeling was carried out at room temperature with a 1/50 dilution of mouse anti HIV-1 p24 mAb (Santacrutz, USA). The secondary staining was performed with PE-conjugated anti mouse polyclonal antibody (Santacrutz, USA).

### cDNA synthesis and PCR

Subsequent to various treatments of immDCs for 24 hrs at 37°C, the total cellular RNA from 2×10^5^ cells was isolated by Total RNA easy kit (Qiagen, USA) following the manufacturer's protocol. 10 µl of the eluted RNA was subjected to reverse transcription by the M-MLV Reverse Transcriptase RNase H Minus (Invitrogen, USA), at 40°C for 60 min with random hexamer (Applied Biosystems, USA) in a total volume of 25 µl. 3 µl of RT product was used as the template for the first round of PCR. The PCR reaction was carried out in a final volume of 50 µl containing 20 mM Tris-HCl (pH 8.4), 50 mM KCl, 1.5 mM MgCl_2_, 0.2 mM each deoxynucleoside triphosphate, 0.2 µM of each specific primer, 2.5 U of ampliTaq DNA polymerase (Applied Biosystems, USA). The reverse transcription and PCR amplifications were carried out with appropriate negative controls to detect any possible contamination during the experiments.

PCR amplification was performed with cDNA using primer pairs specific for IL-6, (upstream primer 5′-GAGAAAGGAGACATGTAACAAGAGT-3′; downstream primer 5′-GCGCAGAATGAGATGAGTTGT-3′), IL-10 (upstream primer 5′-ATGCCCCAAGCTGAGAACCAAGACCCA-3′; downstream primer 5′-TCTCAAGGGGCTGG GTCAGCTATCCCA-3′), CD4 (upstream primer 5′-GCAGAGCGG ATGTCTCAGAT-3′; downstream primer 5′-CTACATTCATCTGGTCCGCAG-3′), CCR5 (upstream primer 5′-CAAAAAGAAGGTCTTCATTACACC-3′; downstream primer 5′-CCTGTGCCTCTTCTTCTCATTTCG-3′, DC-SIGN (upstream primer 5′-GCCACCCCTGTCCCTGGGAATG-3′; downstream primer 5′-TAAAGGTCGAAGGATGGAGAGAAG-3′, SOCS-1 (upstream primer, 5′-GAGAG CTTCG ACTGC CTCTT-3′; downstream primer, 5′-AGGTA GGAGG TGCGA GTTCA-3′), SOCS-3 (upstream primer, 5′-CTCAA GACCT TCAGC TCCAA-3′; downstream primer, 5′-TTCTC ATAGG AGTCC AGGTG-3′) and GAPDH (upstream primer, 5′-TGATG ACATC AAGAA GGTGG-3′; downstream primer, 5′-TTACT CCTTG GAGGC CATGT-3′). The predicted products for IL-6, IL-10, CD4, CCR5, DC-SIGN, SOCS-1, SOCS-3, and GAPDH were 170, 352, 175, 189, 260, 562, 554, and 244 bp respectively. The PCR products were separated on a 2% agarose gel and visualized by ethidium bromide staining.

### RNA interference

immDCs were transfected with upto 100 nM siRNA with transfection reagents DF4 (Dharmacon, USA), according to the manufacturer' protocol. The siRNAs used were: DC-SIGN siRNA, CD4 siRNA, CCR5 siRNA, NF-êB p65 siRNA (Santacrutz Biotechnology, USA), Raf-1, SOCS-1, SOCS-3 and IL-10 SMARTpool; nontargeting siRNA pool, as a control (Dharmacon, USA). This protocol resulted in nearly 100% transfection efficiency as determined by flow cytometry of cells transfected with siGLO-RISC free-siRNA (Dharmacon, USA). At 72 hrs after transfection, cells were used for experiments. Silenced expression of Raf-1, SOCS-3, SOCS-1, IL-10 and GAPDH were confirmed at the mRNA level by quantitative real-time PCR.

### Ras pull-down assay

Precipitation of active GTP-bound Ras from cell lysates was performed with Raf-RBD-GST (Upstate Biotechnology, USA) as previously described [50]. This was followed by SDS-PAGE and immunoblotting with anti-Ras antibody (BD Bioscience, USA). Total Ras was detected in the cell lysates to confirm equal amounts.

### Raf-1 phosphorylation assay

Raf-1 phosphorylation was measured by immunoprecipitation of immDCs with anti-Raf-1 (Upstate Biotechnology, USA) followed by immunoblotting with anti-phospho-Raf (Ser338) (Cell Signaling Technologies, USA). Phosphorylation at ser338 residue of Raf-1 was also confirmed by flow cytometry. The cells underwent a stimulation of 15 minutes with gp120, Nef or both before carrying out the experimental procedure.

### NF-êB phosphorylation assay

Immature monocyte derived DCs nuclear extracts were prepared with the NucBuster protein extraction kit (Novagen, USA), and 5 µg of nuclear extract was used to determine the specific subunits within the DNA binding NF-êB dimers with the TransAM NF-êB family kit (Active Motif, Carlsbad, CA), according to the manufacturer's protocol. The p65 subunits from cell lysates was captured with the Pathscan phospho-NF-êB p65 (Ser536) sandwich ELISA kit (Cell Signaling Technologies, USA), according to the manufacturer's protocol. Total p65 was detected with rabbit anti-p65 pAbs (Active Motif, Carlsbad, CA), p65 phosphorylated at Ser536 was detected with rabbit anti-phospho-p65 (Ser536) pAbs (Cell Signaling Technologies, USA), and acetylated p65 was detected with rabbit anti-acetyl-lysine pAbs (Upstate Biotechnology, USA).

### Immunoprecipitation

Immature monocyte derived DCs cells were washed twice with PBS and solubilized in 1 ml of lysis buffer (0.5% Nonidet P-40, 50 mM Tris-HCl (pH 7.4), 150 mM NaCl, 1 mM NaF, 1 mM EDTA, 1 mM Na_3_VO_4_, 0.25 mM PMSF, 5 µg/ml aprotinin, 1 µg/ml leupeptin, 1 µg/ml pepstatin, and 15% glycerol) for 30 min at 4°C. Insoluble material was removed by centrifugation, and the cell lysates were incubated with specific antibodies anti-Ras (BD Bioscience, USA); anti-Raf-1 (Upstate Biotechnology, USA); anti-STAT3 (Santa Cruz Biotechnology, USA) overnight at 4°C. The immune complexes were bound to protein A-Sepharose (2.5 mg/ml in lysis buffer) for 1 h at 4°C. After centrifugation, the Sepharose beads were washed three times with washing buffer (0.05% Nonidet P-40, 50 mM Tris-HCl (pH 7.4), 100 mM NaCl, 1 mM NaF, 1 mM EDTA, 1 mM Na_3_VO_4_, and 15% glycerol). The samples were boiled in gel electrophoresis sample buffer; the precipitated proteins were separated on SDS-polyacrylamide gels (7.5% acrylamide), transferred to nitrocellulose membranes and probed with appropriate antibodies.

### Immunoblotting and immunodetection

The electrophoretically separated proteins were transferred to a nitrocellulose membrane by the Western blotting method. Nonspecific binding sites were blocked with 10% BSA in TBS-N (20 mM Tris-HCl (pH 7.4), 137 mM NaCl, and 0.1% Nonidet P-40) for 15 min. The blots were incubated with the respective primary Abs in TBS-N for 1 h at room temperature. Primary antibody incubation was followed by incubation with horseradish peroxidase-conjugated secondary antibodies (Santa Cruz Biotechnology, USA) and detection by enhanced chemiluminescence kit (Millipore, USA) was performed during immunoblot analysis. The antibodies used were anti-Ras (BD Bioscience, USA), anti- NF-êB p65, p50, p52, Rel-B, c-Rel antibodies (Santa Cruz Biotechnology, USA) anti-Raf-1 (Upstate Biotechnology, USA), anti-phospho-Raf (Ser338) (Cell Signaling Technology, USA), anti-STAT3 (Santa Cruz Biotechnology, USA) and phosphotyrosine-specific STAT3 (p-Tyr-705) (Santacrutz Biotechnology, USA). Anti-â-actin (BD Biosciences, USA) served as an internal control. Molecular weight markers (Bio-Rad Laboratories, USA) were loaded to ensure that proteins of interest were at the appropriate position.

### Flow cytometry

The expression of DC-SIGN, CD4 and CCR5 on the cell surface of immDCs, phosphorylation of Raf-1 and determination of apoptosis were analysed by FACS. Prior to flow cytometry, the cells were fixed for 10 min in 3% (wt/vol) paraformaldehyde and then permeablized for 10 min at 4°C in 90% (vol/vol) methanol. Viable cell counts were obtained by enumerating cells that excluded trypan blue dye. The antibodies used were anti- DC-SIGN mouse monoclonal, anti- CD4-PE conjugated mouse monoclonal, anti- CCR5 mouse monoclonal (BD Biosciences, USA), anti- phospho-Raf (Ser338) (Cell Signaling Technology, USA). Secondary staining was performed, where applicable, with PE-conjugated secondary antibodies (Santa Cruz Biotechnology, USA).

To detect apoptotic cells, the flow cytometric analysis of propidium iodide (PI)-stained nuclei was performed. In brief, cells were washed with phosphate-buffered saline (PBS) and then incubated in hypotonic lysing buffer (0.1% sodium citrate, 0.3% Nonidet P-40, and 50 µg/mL PI) at 4°C for 30 minutes. Alternatively, cells were stained with annexin V conjugated to phycoerythrin (PE) (BD Bioscience, USA) and vital dye 7-aminoactinomycin D (7-AAD; PharMingen). Cultured cells were washed with PBS, resuspended in binding buffer (10 mM HEPES [N-2-hydroxyethylpiperazine-N′-2-ethanesulfonic acid], 140 mM NaCl, and 2.5 mM CaCl_2_), and incubated with annexin V-PE and 7-AAD (1.25 µg/mL) at room temperature for 15 minutes. Flow cytometry analyses were performed in a flow cytometer (BD FACS ARIA II, USA) by BD FACS Diva Software 6.0.

### ELISA

Cells were incubated with 4 µg/ml gp120 and 100 ng/ml Nef for 24 h. Culture supernatants were harvested, and MIP- alpha, MIP-beta, IL-6, IL-10 and TNF-alpha concentrations were determined by using specific Opt ELISA kit (BD Biosciences, USA).

### Statistical analysis

The results were expressed as the mean±standard error of the mean (SEM), where applicable of three independent experiments. The data were analyzed by Student's t-test. The p values of <0.05 were considered as significant.
