# Correction: HIV-1 Gp120 Protein Downregulates Nef Induced IL-6 Release in Immature Dentritic Cells through Interplay of DC-SIGN

**DOI:** 10.1371/annotation/136a68f8-9038-4124-b7b8-1808d0461c86

**Published:** 2013-10-24

**Authors:** Roni Sarkar, Debashis Mitra, Sekhar Chakrabarti

There were errors introduced during the preparation of this manuscript for publication. The â, ê and ? symbols in the Materials and Methods, Results and Discussion sections of the article are listed incorrectly. The correct symbols for these sections are β, κ and ∆ respectively. 

